# Learning from OCTET – exploring the acceptability of clinical trials management methods

**DOI:** 10.1186/s13063-018-2765-6

**Published:** 2018-07-13

**Authors:** Catherine Arundel, Judith Gellatly

**Affiliations:** 1 Department of Health Sciences, University of York, York, UK; 2 Division of Nursing, Midwifery and Social Work, University of Manchester, Manchester, UK

**Keywords:** Randomised controlled trial, Project management, Trial management, Qualitative

## Abstract

**Background:**

Conducting research can be time consuming, difficult and challenging. Guidance and pragmatic advice focussing on randomised controlled trial conduct are available but do not necessarily constitute comprehensive guidance. A successful trial is one that recruits to time and target and collects high-quality data within the originally agreed budget. Standardised trial management tools have outlined key project management elements for a successful trial as a method of ensuring good practice in research trials: initiation, planning, execution, monitoring and closure. Lessons are also frequently learnt during the development and conduct of trials but rarely shared for the benefit of others.

For the wider research team, the key focus will always be on the execution and delivery of a study. The aim of this study was to evaluate the acceptability of clinical trials management methods, focussing on study execution and monitoring, as implemented in the National Institute for Health Research Health Technology Assessment Programme-funded Obsessive Compulsive Treatment Efficacy Trial (OCTET).

**Methods:**

Workshops, questionnaires and semi-structured interviews were used to explore acceptability of trial management methods with members of the OCTET Trial research team. Nine members participated in the focus group, 10 completed a questionnaire and 20 were interviewed as part of qualitative work for the main OCTET study. Data was collected and analysed using thematic analysis.

**Results:**

Six key themes were identified: support; communication; processes; resources; training and ethos. Clear and open communication, enthusiasm and accessibility of the trial managers and chief investigator were consistently noted as an important facet of the successful running of the trial. Clear resources and training materials were also found to be crucial in helping staff to work within the trial setting. Constructive suggestions were also made for improvement of these resources; for example, including both checklists and flowcharts within trial processes.

**Conclusion:**

Organisation, openness and positivity are crucial for executing a trial successfully, whilst clear and focussed processes and resources are essential in monitoring and controlling the trial progress. Although derived from a single study, these findings are likely to be applicable to the successful conduct of all trials. Trial managers should consider developing these elements when setting up a study.

**Trial registration:**

Clinical Trial Registry, ID: ISRCTN73535163. Registered prospectively on 5 April 2011.

**Electronic supplementary material:**

The online version of this article (10.1186/s13063-018-2765-6) contains supplementary material, which is available to authorized users.

## Background

Conducting research is time consuming and can be difficult and complex for all involved. The varied procedures involved in conducting a randomised controlled trial (RCT), commonly considered as the ‘gold standard’ of research paradigms [1], often make this process even more challenging. Many trials fail due to potential barriers being overlooked, the inability to achieve support from stakeholders or procedures being misunderstood [2, 3]. Lessons are learnt during the development and conduct of a RCT but are not often shared for the benefit of others.

A successful trial can be defined as one that recruits to both time and target, collects high-quality data and completes activity within the originally agreed budget [4]. Good trial management is required in order to ensure that stated objectives, needs and expectations of a trial are met within restricted time frames and budget constraints [4, 5]. The principles of good management are not restricted specifically to research, spanning a variety of professions including financial services, engineering and The military. Increasingly it has been considered necessary to adopt a ‘*business-like approach*’ to the delivery of clinical trials in order to succeed [6, 7].

Project management has been defined as ‘*the application of knowledge, skills, tools and techniques to project activities to meet project requirements*’ ([8] p. 6). In ensuring that quality standards are met in an efficient and punctual manner [9], five processes are considered a vital part in its execution:

1. Initiation – Formal start point of project. The project is broadly defined and the feasibility of proposed research is determined
2. Planning – Comprehensive plan developed addressing goal setting, identification of costs and resources, risk management planning, modes of communication with stakeholders, timelines and ensuring that roles and responsibilities are clearly defined
3. Execution – Development and completion of deliverables to meet the required projects aims and objectives. Allocation of resources and support provided to team members to ensure that assigned tasks are completed
4. Monitoring and controlling – Occurs alongside the execution phase focussing on measuring project progression and performance in line with previously agreed goals and timelines. Identification of strategies to keep the project in line with agreed timelines and deliverables if variation is identified
5. Closure – Formal closure of the project. Collating all documents and deliverables, disseminating findings. Termination of relevant contracts also occurs

Research evidence has suggested that despite the importance of having a well-constructed protocol, success is enhanced by efficient management of research tasks, timelines and staff [10]. It is generally considered that approximately 50% of the total time spent on a study relates to its conduct, including recruitment of participants and collection and monitoring of data [11]. A poorly conducted trial not only impacts upon the potential success of the trial but potentially infringes ethical procedures and the rights of research participants [12]. For example, it may result in incorrect processes being followed with regards patient-informed consent or withdrawal; therefore, conflicting with both ethical principles and participant rights. The responsibility for preventing this ultimately lies with the chief investigator. However, trial managers, by virtue of overseeing the daily running of a trial, are integral in supporting sites with regards to appropriate implementation of trial-specific processes and in ensuring that outputs are delivered in time and to budget [13, 14]. Trial managers have to project confidence amongst other team members, be highly organised, communicate and coordinate effectively, have the ability to multi-task, think laterally and strategically and be motivational [4]. If the trial manager is performing successfully, enhancing communications and negotiations with significant contributors via an appropriate infrastructure [15], trial outcomes can be optimised [10].

In contrast to the abundance of guidance available for designing and conducting research, there is a paucity of academic literature drawing upon expertise to best inform the execution of trials [2, 16]. Anecdotally, it is thought that this may leave many trial managers feeling that they lack direction. The guide to efficient trial management [5], produced on behalf of the National Institute of Health Research (NIHR) Trial Managers Network provides useful general guidance about managing trials, providing pragmatic legal and operational advice and guidance. However, there remains little evidence drawing upon the practicalities of implementing such guidance [17], its impact and acceptability. A few researchers have attempted to gain insight into the experiences of individuals involved with research trials [16, 18] but have presented potentially subjective findings, predominantly retrospective in nature. Furthermore, the views of the diversity of staff involved are not captured and the predominant focus is on the set-up or reporting phases rather than the trial conduct. Irrespective of the aims and objectives, size or focus of an RCT the underlying management procedures will continue to apply and, therefore, useful lessons learnt in any trial can facilitate the design and conduct of any new trial.

## Methods

### Aims and objectives

This paper will draw upon the trial management experiences within a large-scale UK mental health trial. Initially presenting some contextual information about the trial to which it relates and its set-up, feedback from individuals involved in different aspects of the conduct, management and monitoring of the trial will then be presented. This paper aims to provide insight into the experiences of individuals involved in diverse aspects of the conduct of a large RCT and to provide suggestions and guidance on to how best manage such trials. Using prospective data collection approaches it will directly identify key issues, in relation to clinical trials management methods, that should be drawn upon when planning, developing and conducting future research trials. Given the prominence of the trial management role within the execution, monitoring and controlling phases of a trial, feedback will predominantly relate to these processes but may extend into earlier and later aspects of the project processes.

### In context: The Obsessive Compulsive Treatment Efficacy Trial

The National Institute for Health and Care Excellence guidelines for obsessive compulsive disorder recommend that people with obsessive compulsive disorder should receive a stepped-care approach to treatment, similar to that of anxiety and depression [19]. There is, however, little evidence to support the treatment of low-intensity psychological interventions.

The Obsessive Compulsive Treatment Efficacy Trial (OCTET) was a three-arm RCT which aimed to evaluate the effectiveness of two low-intensity interventions – Guided Self-help and Computerised Cognitive Behavioural Therapy – for adults experiencing obsessive compulsive disorder symptoms in comparison to waiting for cognitive behavioural therapy treatment [20]. Study recruitment ran between February 2011 and May 2014, and the study completed, following last patient last visit, in May 2015. The study set out to recruit 432 participants, although this was subsequently increased to 472 to allow for lower than anticipated retention at the primary outcome time point [21]. The trial was multi-sited. Four main university-based research sites in England were set up at the outset and were supported by four NIHR Clinical Research Network (formerly the Mental Health Research Network) sites. In total, 15 NHS Trusts, within NHS England took part in the trial, participants were recruited from 14. Clinic list screening was conducted at each of the NHS Trusts to identify potential participants. Patients were sent information about the study and asked to return a ‘Consent to Contact’ form to express an interest in participation. On receipt, the local site research staff contacted the participant to arrange a consent, assessment and randomisation visit. Occasionally a delay was incurred between receipt of a ‘Consent to Contact’ form and an assessment visit being completed due to researcher and/or participant availability. Following randomisation, participants were allocated to a psychological wellbeing practitioner based in primary care mental health services. The psychological wellbeing practitioners were responsible for providing support for the low-intensity obsessive compulsive disorder interventions, developed for the trial, which were expected to be delivered over a 12-week period. Individual psychological wellbeing practitioners were automatically allocated to a participant by the OCTET management database. Where the allocation was not accepted within 72 h, a trial manager made contact with all local psychological wellbeing practitioners by email to request acceptance of a participant for support.

The primary outcome for OCTET was the Yale Brown Obsessive Compulsive Scale (YBOCS)-Observer Rated as collected at the 3-month follow-up visit. Methods to ensure blinded outcome assessment included ensuring that study follow-up was completed separately to intervention delivery and by restricting researcher access to group allocation information in the trial management database [21]. Blinding was monitored throughout the trial and, where research staff were unblinded, every effort was made to ensure continued blinded outcome assessment by completion of follow-up visits by another, independent and blinded assessor [21].

OCTET met the definition of a successful trial as it recruited to time, exceeded target by virtue of achieving the revised sample size and was completed within the specified budget for the study. Overall, the study obtained high-quality data, achieving 80% completion of the designated primary outcome (YBOCS-Observer Rated or its proxy, the YBOCS-Self-Rated). It is, however, worth nothing that the economic data collected for OCTET was compounded by missingness occurring when participants were unable to complete comprehensive follow-up [21]. Given that the study results remained robust to imputation to deal with this missingness, the integrity of this as a successful trial is not affected.

#### The Trial Management Team

During the design of OCTET, it was intended that two trial managers would be appointed to the trial, one based in the lead site and the other in the Clinical Trials Unit supporting data collection. Two trial managers, with previous health services research experience and experience of managing trials, were subsequently appointed and distinct roles with defined responsibilities were established; however, cross-over between some responsibilities was acknowledged. The trial manager in the lead site was responsible for the training and support of research staff, supporting clinical staff involved in treatment delivery and the day-to-day running of the trial, whilst the other led the management and monitoring of data collection, retention rates and adverse event reporting. The trial managers provided cover for each other when required. Both trial managers undertook routine monitoring of study deliverables including recruitment and retention rates, time taken to contact participants, and serious adverse event reporting. This routine monitoring formed the basis for formal reporting to wider groups responsible for monitoring study conduct such as the Data Monitoring and Ethics Committee, the Trial Steering Committee and the funder.

Numerous other individuals were involved in the conduct and management of the trial. These included academics, clinicians, statisticians and health economists who formed the Trial Management Group and the study chief investigator all of whom provided input to the management of the trial.

The Trial Steering Committee and Data Monitoring Committee provided independent support, advising on quality and scientific aspects of the study, providing direction when needed. These committees also formed part of the formal monitoring for the study with Terms of Reference generated and agreed prior to study activity commencing. Patient and Public Involvement representatives were members of the Trial Management Group and Trial Steering Committee.

Research staff (research assistants and clinical studies officers from participating NIHR Clinical Research Network sites) were responsible for the recruitment and follow-up of research participants at the study sites. Psychological wellbeing practitioners, based within Improving Access to Psychological Services teams, were responsible for the delivery of the intervention. There was no cross-over between the roles allocated to these two groups.

Clinicians at Trust sites also provided supervisory support to clinical staff delivering trial treatments and administrative staff assisted with recruitment.

In addition to the provision of one of the trial managers, the registered Clinical Trials Unit (York Trials Unit – Reference: 40) also provided support to OCTET specifically in relation to data management including provision of the study database and the secure, central randomisation service.

In overseeing the progress of the trial, ensuring good working practices and to support research staff working on the trial the trial managers developed a variety of procedures, systems and resources.

#### Training

Extensive training was provided for research staff involved in recruitment and conducting baseline and follow-up research visits (research assistants and clinical studies officers). Training, delivered in a group setting over 1 day by one of the trial managers, involved ensuring that research staff were familiar with all of the trial procedures and outcome measures used for data collection. Following the training the rating of an example primary outcome interview (YBOCS) was required for inter-rater reliability purposes. This task was repeated 6 months later. Where research staff joined the team during the trial, training was provided on an ad-hoc basis in a group setting or individually as appropriate.

Psychological wellbeing practitioners involved in supporting participants with the trial interventions also received training delivered over 3 days – 2 days for Guided Self-help and 1 day for Computerised Cognitive Behavioural Therapy. The training was delivered by the chief investigator, co-applicants and representatives from computerised cognitive behavioural therapy (CCBT) Ltd. in a group setting and focussed on familiarising psychological wellbeing practitioners with the Guided Self-help workbook and Computerised Cognitive Behavioural Therapy programme. A variety of training methods were utilised including small- and large-group work and skills practice with specific feedback using hypothetical but typical cases of moderate and severe obsessive compulsive disorder. Training manuals were provided for both treatment arms. Where psychological wellbeing practitioners joined the team during the trial, training was provided on an ad-hoc basis in a group setting to ensure that there was continued availability of practitioners in all recruiting locations.

#### Trial-specific procedures

Trial-specific procedures were generated by the trial managers, in conjunction with the wider Trial Management Team, for all aspects of the study to support research staff and psychological wellbeing practitioners. Researcher trial-specific procedures focussed on recruitment procedures, the conduct of participant assessments and follow-ups, retention, reporting risk and adverse events and managing distress. Psychological wellbeing practitioner trial-specific procedures provided contextual trial information including recruitment procedures, detailed randomisation processes, outlined recording and storage of intervention session procedures and monitoring practices.

A delegation log was produced and agreed formally by all trial individuals to ensure that all expected roles were observed. Those persons added to the delegation log were confirmed to be competent in completing study processes by virtue of having completed associated study training.

### Data collection

To evaluate the acceptability of clinical trials management methods, feedback from individuals involved in the trial was sought using three methods:

1. During a researcher focus group (RFG) that took place at the end of the study recruitment period. Comments were provided verbally, recorded in meeting minutes by one of the trial managers and subsequently ratified by attendees. An interactive exercise was also used, where participants were asked to provide comments anonymously on Post It notes for each key question. Aspects of the trial discussed included management, procedures, training, their involvement and experience, what they thought had gone well and what could have been better
2. Completion of a ‘Learning from your experiences’ questionnaire sent to research staff who were unable to attend the RFG and other individuals involved in the trial (e.g. Data Monitoring and Ethics Committee, Trial Steering Committee and Trial Management Group members and site leads) (Additional file 1). This questionnaire collected qualitative information on trial team member’s experiences of participation in OCTET, specifically, what they thought had gone well and not so well, if training needs were met, what aspects of the trial could have been organised or conducted differently to make it more efficient and their experience to be more positive and how their experience differed from working on other research trials and working environments
3. Semi-structured qualitative Interviews with psychological wellbeing practitioners were conducted by telephone as part of the trial acceptability evaluation by a psychological wellbeing practitioner employed as a member of the trial team. A proportion of the topic guide specifically explored their experiences of being involved in the trial and other discussions held gave rise to their views of delivering treatment as part of a research trial. Interviews were conducted with psychological wellbeing practitioners who had supported at least one patient in the trial. As this data was not reported elsewhere, it was included in this study, alongside data collected from other groups, via different methods

Individuals were encouraged to be as honest as possible when answering the questions highlighting positive and negative aspects of their involvement, but given the nature of data collection, responses were not anonymous.

### Analysis

Focus group notes, questionnaire free-text and interview transcripts were collated. Psychological wellbeing practitioner interviews were audio-recorded with verbal participant consent and transcribed verbatim. NVivo qualitative data analysis software version 10 (QSR International, Melbourne, VIC, Australia) was used to assist with the management of the interview data. Questionnaire and focus group data were managed using Microsoft Excel. All data was analysed using thematic analysis [22]. Data from the different sources was reviewed by the OCTET Trial managers and compared in order to identify commonalities and differences between individual experiences, and opinions about their role within the trial and experience of its management. The two trial managers independently coded the data and met to discuss their personal interpretations of the data to review and reflect in order to agree on a final coding structure.

## Results

Thirty-nine individuals involved in differing roles within the trial provided feedback about their experiences of the acceptability of clinical trials management methods used in OCTET.

- Twenty-five research staff members (research assistants and clinical studies officers) were invited to attend a researcher meeting of which a focus group exploring trial experiences formed an element. Nine researchers, representing seven participating sites, attended and provided feedback
- Thirty-two members of the OCTET research team (including researcher, site leads and Data Monitoring and Ethics Committee and Trial Steering Committee members including Patient and Public representatives) were approached by email and asked to complete the ‘Learning from your experiences‘ questionnaire (Additional file 1). Ten members provided feedback including three researchers, three Trial Steering Committee members, two Data Monitoring and Ethics Committee members and two site leads
- Seventy psychological wellbeing practitioners were offered the option to participate in a trial qualitative interview. Twenty participants, representing 11 NHS Trusts, responded, all of whom completed an interview

## Findings

Six main themes were derived from the data, based on the experience of the OCTET team, as an evaluation of the trial organisation and as learning points for future trials. For the purposes of reporting, the following coding has been used to identify the type of respondent and/or response method associated with the data: PWP – Psychological wellbeing practitioner interview); RFG – Researcher focus group; DMEC/TSC – DMEC or TSC member questionnaire; R – Researcher questionnaire; SL – Site lead questionnaire.

### Theme 1: support

The study team reported that trial support was both positive and effective. It was felt that the trial team collectively provided seamless multi-disciplinary working which fostered a feeling amongst both researcher staff and psychological wellbeing practitioners that they were valued and could seek support as required:

‘It was quite plain that the support was there and the encouragement to make a success of the trial was there.’ (PWP)

‘The trial managers were approachable…I could talk about any issue or admit if I was struggling with things.’ (RFG1)

Support in relation to researcher safety and care was highlighted as extremely important to research staff. In particular, the importance of being able to contact a member of trial management promptly to discuss safety concerns was enhanced by having dedicated trial managers who could be contacted easily:

‘I knew I could leave an interview if I felt unsafe at any time.’ (RFG2)

‘Team support has been great…usually it’s just the clinical lead or PI, however, having dedicated trial manager support meant I could get a response to my query quickly.’ (RFG3)

Routine meetings were held during the trial, with dedicated meetings to support the study researcher staff. Due to the distribution of sites across the UK, face-to-face meetings were limited and sites noted that this could have been improved by rotating meeting locations. Independent committee members also noted this, and the frequency of meetings, as a point for future consideration:

‘Trying to stay up to date with such infrequent meetings may be easier for the chair as they have more regular contact.’ (DMEC/TSC1)

‘It would have been appreciated if some of the researcher meetings could have been held around the country.’ (R3)

Psychological wellbeing practitioners felt that supervision, provided by members of the Trial Management Team (i.e. the chief investigator and clinical site leads), incorporated tailored expertise and encouragement and so supported learning development. Consistency and reliability of supervision was noted to be important and this was often attributed to the feeling that the chief investigator, or site leads, believed in the work of the psychological wellbeing practitioners. It was, however, noted that the method of supervision delivery sometimes limited the ability to take full advantage of the support available. In addition, there were mixed views on the frequency of supervision and the impact this had on delivering both trial and clinical commitments:

‘I felt like I knew what I was doing; I felt like I had enough support, I felt that I could get more support if I needed to.’ (PWP40)

‘I suppose sometimes the problem of getting the time to do the supervision, that was the only problem… It did sometimes, and I’ll be honest, feel a bit like I’m working really hard anyway, I don’t need this on top.’ (PWP37)

‘It’s always difficult having supervision over the phone or with someone you’ve never had supervision with before…’ (PWP54)

‘Obviously you’ve got to be aware of the service you’re within as well as the trial…I had to also advise my other supervisor and managers…’ (PWP43)

### Theme 2: communication

Communication was rated positively by all members of the study team, in particular, the personable natures of the trial managers which helped to motivate and encourage the wider trial team. This is perhaps a ‘*“soft factor” which should not be underestimated’* (DMEC/TSC3) particularly as such personalities were felt by the chief investigator to have enhanced the reputation of the trial, both internally and externally:

‘Having that external face of OCTET (i.e. great emails and always being so helpful) enhanced the reputation of the trial.’ (SL1)

Researcher staff noted that the way a message was phrased, impacted upon their perception of the information conveyed. The variety of communication pathways, that these were always available, and the prompt responses provided to queries, is also likely to have been conducive to fostering feelings of support as described in theme 1:

‘If it seemed important to the trial managers, this made the information important to the researchers.’ (RFG8)

‘If I have emailed people, emailed me back within 15, 20 minutes. People have always said when they’re going to be off.’ (PWP71)

The timeliness of communication was of particular importance to committee members who noted the inclusion of both visual and written documentation, which was prepared for discussion in trial meetings:

‘The PowerPoint in preparation for the meetings was very good and was not expected.’ (DMEC/TSC1; DMEC/TSC4)

### Theme 3: processes

The data collection process used in OCTET minimised reliance, seen in other trials, on clinical staff to collect or provide trial data. A study retention policy further supported data collection, allowing researcher staff to work independently to plan visits with ongoing oversite from the trial managers.

‘I was also aware that the completion of visits was being monitored – I actually found this helpful as sometimes cases could potentially get overlooked.’ (R1)

Research staff did, however, note that unblinding had been problematic, particularly in interviews with longer duration. In longer interviews participants inadvertently, in conversation, discussed their treatment allocation with the research, despite being reminded not to discuss this at the start of the visit. Additional interview training and guidance could be an easy strategy to help to minimise unblinding in future RCTs which would in turn ensure ‘continuity of care’ for study participants:

‘Unblinding has been a difficulty throughout the trial and this seemed to increase the longer the visit duration.’ (RFG9)

‘Whilst swapping to different staff after unblinding could help, continuity of care may also be beneficial.’ (RFG10)

Psychological wellbeing practitioners gave a mixed review of the intervention allocation processes used. Some felt that allocation should have been to a specific individual rather than to all practitioners. This highlighted a misunderstanding of the procedure as psychological wellbeing practitioners were approached individually with a group request only if a participant was not picked up. A further suggestion was made that a centralised psychological wellbeing practitioner should have been in place to take on unallocated participants:

‘I think the allocations, possibly that system could have been improved. At the start I think people were allocated but then it seemed that we’d just email everybody and then somebody would pick it up and I think it was that general feeling of, oh, somebody else will do it.’ (PWP19)

Psychological Wellbeing Practitioners noted that there sometimes were delays between patient referral to OCTET and randomisation for the trial. Every effort was made to minimise this delay; however, scheduling difficulties and researcher availability may have impacted in some instances:

‘The main thing is actually the time between someone opting in for treatment or consenting to contact and then randomisation…That takes quite a lot of time…It can go months without us hearing anything, which is quite disconcerting if someone is still on your caseload.’ (PWP54)

### Theme 4: resources

Study resources were found to be sufficiently detailed, clear and easy to use. For some, however, the number of revisions sometimes made it difficult for sites to maintain version control. However, it is important to note that this did not result in any protocol deviations or violations. The addition of checklists to supplement procedures was found to be useful and it was suggested that flowcharts would also have helped implementation of trial procedures:

‘Patients…have appreciated by proxy the care and attention that has gone into the SOPs and study procedures.’ (R3)

‘The checklists for the visits were really useful…flow charts could also be used to supplement these.’ (RFG11)

‘Fewer SOP revisions…lots of paperwork.’ (R3)

Psychological wellbeing practitioners noted that having additional intervention packs in stock, to prevent delays in providing this to participants, would have been useful. However, the availability of the trial managers to respond to requests limited this from being a substantial issue:

‘I’ve requested an extra booklet…and they said “oh!, just give us the number and we’ll send it straight to them” and they got it a day later.’ (PWP71)

Building links with the local service was noted to be important in facilitating set-up and organisation of the trial. It was not possible to implement this effectively at all study sites, which may have limited local service engagement across the trial. In addition, clarification of expectations of all involved groups, from the outset, was noted by as being important. This was, however, easily rectified through clear communication as the trial progressed:

‘The service has been great and the additional work we did on building relationships and getting to know the clinical teams was important in this process.’ (SL2)

‘We should have been less CTU naïve as we were unclear about what they were providing which led to initial tensions.’ (SL1)

Many research staff members commented on the study database and indicated that improvements could have been made to help to facilitate ease of use and study coordination. This included addition of methods to track follow-up visits, limitation on database time-out and hiding of withdrawn participants from the study case lists:

‘The database has been difficult to navigate as the lay out is not intuitive…this is, however, better than some…simple can be better.’ (RFG5)

‘An ability to track follow-ups and see what’s due and when… would be particularly helpful if sites include multiple researchers as this proved difficult to coordinate at times.’ (RFG6)

‘Withdrawn cases remained visible…and showed up as appointments due. This was confusing when working out which visits did actually need completing.’ (RFG12)

### Theme 5: training

OCTET training was positively received by both psychological wellbeing practitioners and research staff. Face-to-face training was felt to be most beneficial; however, teleconference training, conducted by the site lead, was also well received. Shadowing of research interview visits was offered as an additional training opportunity for research staff, with new research staff observing interviews conducted by experienced OCTET researchers. This provided a further training opportunity and included additional support and encouragement prior to completing interviews independently:

‘It was well structured and came away from session feeling very positive. I found…phone-in session very innovative.’ (R2)

‘Shadowing visits was very helpful.’ (RFG7)

Training, coupled with the materials and resources, provided confidence in working to trial procedures. Both psychological wellbeing practitioners and research staff suggested that additional role-play opportunities would have further improved the training; for psychological wellbeing practitioners focussing on complex obsessive compulsive disorder presentations, for research staff focussing on handling patient experiences and personal safety:

‘Practical experience, e.g. role play would be helpful to consolidate learning.’ (RFG13)

‘It would be good to have some role play, or some experience of managing more difficult assessments…’ (PWP25)

Research staff also suggested that training should be tailored to past experiences, i.e. intensive training for those without associated clinical experience and a reduced training session for those with a clinical background. This, coupled with a session with a clinician, would have helped some research staff to feel more confident in completing the YBOCS [23] tool during initial study visits:

‘I felt the training provided a good basis to build on and I understood the principles of how the measure should be scored but I found it more difficult undertaking YBOCs initially as I have never worked with this client group before...It may have been helpful to have had group meetings for RAs initially to discuss ratings of YBOCs interviews and to have the opportunity to discuss with a clinician...’ (R1)

Training in trial procedures and processes, was offered on a frequent basis throughout the trial duration. Despite the number of training sessions offered, enthusiasm was evident at each training session and prompted trainees to reflect and develop their own learning:

‘It wasn’t just delivered; it was engagement and discovery of how it was going to be done. And their passion for it as well came across.’ (PWP37)

‘It opened by mind to it. It gave me some new questions to think about, and there was someone with expertise in it to ask questions and develop my learning.’ (PWP43)

It was suggested that provision of refresher training for psychological wellbeing practitioners who were delivering the trial intervention, would have been a useful addition, due to the gap sometimes experienced between training and taking on initial patients for the trial interventions:

‘The big gap meant I was having to go through everything again… if I’d got a patient the next week I would have been absolutely fine, it would have been very fresh in my mind.’ (PWP59)

‘The training was good and maybe if it was to continue, a bit of a refresher might be good.’ (PWP37)

### Theme 6: ethos

The Trial Committees noted that the team were enthusiastic, motivated, respectful and open to suggestions for improvement, with particular note made to the difficulties experienced, but overcome, with regards changes in NHS referral pathways resulting in slow recruitment and the need to increase study sample size to accommodate lower than expected retention (expected 78% rather than 85%):

‘The trial team have snatched triumph out of the jaws of disaster.’ (DMEC/TSC2)

The dedication and enthusiasm of the trial team to make the study work was also noted, with members of the wider study team feeling encouraged, as a result, to make the trial a success:

‘The ability to motivate and encourage others at distant sites was inspirational…even when times were harder there was always a feeling of optimism.’ (DMEC/TSC4)

Many psychological wellbeing practitioners and researcher staff reported that they had enjoyed their experiences within the study, were pleased to have been able to be involved, and some would have been keen to become more involved in the study given the opportunity:

‘From the start of my involvement I have felt a valued team member of the study…’ (R2)

‘I think it was fantastic to be a part of, and I was really grateful to be able to be involved in it. I would have really liked to have been more involved or having more cases …’ (PWP48)

Whilst some enjoyed the move into work focussed on obsessive compulsive disorder, one psychological wellbeing practitioner indicated that they had found that this change brought about some personal anxieties. These were, however, reported to have been helpful in challenging the practitioner, ultimately resulting in a positive feeling about this type of work and bringing about a realisation that short-term interventions can in fact be effective for some:

‘This has challenged my anxiety about taking on new things and working with people with different experiences. It has challenged my anxieties about myself and how I would cope. It’s challenged me on different levels and it has challenged my perceptions about sometimes some difficulties, deep underlying work and all this…there are things that can be helpful as a short term intervention that can get people moving quite a lot. And I always find myself pleased when that happens as well.’ (PWP71)

## Discussion

This study explores the acceptability of clinical trials management, from the perspectives of a wide range of individuals involved in the delivery of a single, large, multi-centre study (Obsessive Compulsive Treatment Efficacy Trial – OCTET), focussing specifically on elements relating to the execution (e.g. study delivery, resources, support) and monitoring (e.g. project progression, performance and strategy) of the study.

Within the six main themes arising from the data, key elements spanning all themes were identified as important for the effective execution and monitoring of this study. Given the findings are derived from a wide range of professionals and research staff involved in a large, multi-centre study, many of the findings are likely to apply to other research trials of both mental and physical health conditions.

Clear, open, positive, but focussed communication, through a variety of communication pathways, was noted as crucial to successful execution and monitoring of the study, as were prompt responses to queries. Undoubtedly, the inclusion of two trial managers in this study helped to ensure that there were clear and prompt communication pathways at all times. This study has provided evidence of the importance of trial managers having a friendly, personable nature as a method of helping to forge bonds between the trial team, which can be critical to the successful management of the trial. This builds on the work by Farrell et al. (2010) who suggested that appropriate communication, through a variety of methods, helps to ensure that team members feel sufficiently valued and so maintain trial engagement [2]. The findings from OCTET have identified methods by which this can be conducted within a trial setting to promote the successful conduct and completion of the trial. Maintaining a feeling of value, and so engagement, is critical to ensuring that output improved through consistent quality of collected data and that sufficient quantity is collected to facilitate trial analysis [24]. These factors in combination are the cornerstones of trial success and would undoubtedly apply across all research methodologies, settings and topic areas.

Farrell et al. (2010) noted that continuing to promote a positive trial image ensured continued engagement of study researchers and sites [2]. OCTET has provided further evidence of the importance of positive trial image in promoting engagement of study researchers and sites; enthusiasm and positivity from the trial managers and chief investigator from the outset at study training and right through the trial, was seen to be effective at encouraging the wider research team to support the trial. As OCTET included screening and enrolment of patients through NHS services, positive communication was also crucial in building links with service providers. For trials using a similar structure and where input and engagement from a range of services is crucial to support trial activity, the findings here should be able to be generalised across settings. Unfortunately, in OCTET, strong links could not be established and/or maintained with all involved services for the full duration of the trial. This was partly due to differences, and changes, in service structure and service staffing during the trial (including multiple staffing changes and changes to waiting list management which impacted on the levels of engagement and the ability of services to recruit in some settings). Had communication pathways been established with all NHS Trusts involved from the outset it is likely that these links would have been successfully forged.

Irrespective of the time elapsed since Farrell (1998) indicated that ‘*robust systems and procedures must be designed that are efficient, effective and flexible’* ([7] p. 1236); these factors continue to be of high importance to researchers working on RCTs. The findings from OCTET emphasise the importance of the provision of clear and focussed procedures and resources as important to both trial execution and monitoring, and has identified suggestions of how this can be completed. As part of this study, research staff noted that a variety of procedure and training documents (e.g. in written, face-to-face, video and flowchart formats) helped to clearly convey the key elements of the study. It was suggested that procedures and training could be improved by tailoring these to fit different levels of expertise and roles within the research group. The provision of robust procedures will be applicable across all trial types and settings. Depending on the quorum of staff involved in study activity, and their levels of expertise with both the condition of interest, and research activity more broadly, development of procedures to fit with differing levels of expertise may also be appropriate. Given the number of documents required for OCTET, some sites identified that it was difficult to maintain version control. Development of processes to confirm receipt and filing of revised documentation by study teams, or provision of an easily accessible, and up-to-date, version control log may help to alleviate this in future, large, multi-centre trials.

Research staff also noted important improvements which could have been made to the OCTET database; tracking follow-ups, hiding withdrawn patients, reducing automated time-out period. User testing of the database was completed by the trial managers prior to roll out; however, the input of researchers was not considered as part of this. Including researchers in the design and testing of systems may, therefore, improve functionality both within, and across, trials.

Of particular note in relation to procedures and resources, was the inclusion of robust safety procedures (i.e. a telephone buddying system) in addition to local employer lone-working arrangements. Patient safety is frequently considered during study set-up and execution; however, researcher safety is not always given the same consideration within the context of trial procedures (despite both being of equal importance) and there is a paucity of academic literature in relation to this in the context of trial management. Establishing clear within-trial processes for researcher safety, to supplement or support local lone working policies, is particularly important, particularly when face-to-face visits for trials are often conducted outside of NHS settings. OCTET required face-to-face follow-up, at a setting convenient to the participant. The focus on researcher safety may not, therefore, apply to all study settings, but is likely to apply to similar trials (of both mental and physical health conditions) where face-to-face visits are required.

The findings from OCTET fit with suggestions on effective trial management methods highlighted in previous research [2, 7]. The results of this study present evidence of how effective trial management can be achieved and are likely to be generalisable to the conduct of other clinical trials utilising similar trial management frameworks; for example, studies with shared trial management activity (e.g. where coordination is split between the lead research site and a registered Clinical Trials Unit) or where multiple groups are involved in distinct elements of study delivery (e.g. clinical staff delivering interventions, university research staff conducting data collection). Many of the key elements identified are, however, likely to be generalisable beyond these individual settings to most, if not all, trials given that they focus on principles central to, and largely utilised across all trial designs and settings (Additional file 2).

## Research limitations

This study was conducted through focus groups, interviews and qualitative questionnaires which were coordinated by the OCTET Trial managers. As the data collection was not, therefore, independent of the management team, this may have limited the honesty of responses provided. Individuals were encouraged to be as honest as possible when answering the questions and it was emphasised that both positives and negatives were important to report. Individuals were aware that providing responses was for the purposes of trial management development only and would have no bearing on current or future employment, given this was not a responsibility of the trial managers.

Given the nature of data collection, responses were not anonymous which may have further impacted upon the honesty of responses provided. As both positives and negatives were reported as part of data collection, the impact of data collection on respondent honesty is unlikely to have been significant; however, it is noted that the number of positive comments reported outweighed the negative comments. The limited numbers of negative comments were likely to be due to implementation of feedback mechanisms throughout the study (e.g. regular meetings with research staff) which enabled continued feedback, reflection and revision of trial processes to resolve any issues identified by the trial team. For example, during the study the burden of adverse event reporting (i.e. reporting every untoward medical event) was identified as a concern amongst research staff given the number of events reported which were in no way related to the trial (e.g. colds, broken bones). Following review of event reporting, and discussion with clinical members of the team the adverse event procedure was reduced and refined to require only those events relating to trial involvement or the condition of interest to be reported. In addition, the trial managers worked to ensure that core study principles (e.g. timely contact with participants, high levels of recruitment and retention, compliance with study processes) were maintained across study sites but with necessary adaptions, when required, to ensure that procedures were easily and effectively implemented within local NHS Trust policies and structures.

Those who responded to requests to participate in study feedback may have had differing motivations for participation and, therefore, may not necessarily represent the collective views of the full study team. A response was, however, provided by a representative from each study site and, therefore, any site-specific issues are likely to have been captured within this review. Furthermore, where data was collected through questionnaires, it was not possible to fully explore specific comments, which limited the depth of information available. Future research in this area should, therefore, consider using either semi-structured interviews only, detailed questionnaires to enable a depth of information to be captured, or a combination of questionnaire followed by interview, both of which would help to facilitate further exploration of responses.

The interview data collected with psychological wellbeing practitioners included only those who had supported more than one participant with the trial intervention. Some practitioners will invariably have had more experience than others; for example, the number of participants supported and/or may have had different amounts of experience in delivering the two study interventions. The differing levels of involvement are likely to have influenced overall experiences and may have generated some bias, by virtue of varied activity levels resulting in varied levels of exposure to trial procedures, which may affect the generalisability of the findings. As responses were, however, provided by representatives with a range of experiences any specific issues are likely to have been captured within this review.

Data collection was completed following the end of study recruitment and so views and comments associated with study follow-up were not fully captured in the data set. Whilst it is unlikely that comments would have changed as the study progressed, given that follow-up was already in progress, the lack of data from the follow-up period limits the findings somewhat.

Analysis of the responses was completed by the two trial managers to ensure that there were no discrepancies regarding interpretation of responses. Should interpretation have been unclear, input from a third party or the participant themselves would have been sought; however, this was not required during the analysis of findings.

As the two trial managers were involved in analysing responses, this may have introduced bias in the interpretation and reporting of the findings. This could have been rectified through independent analysis and interpretation however, this was viewed as a learning opportunity for the trial managers and it was agreed a priori that both positive and negative comments would be viewed with equal importance. Whilst results may have been different if independent analysis was conducted, the transparency of results reporting, including both positive and negative comments is likely to limit the impact of this.

## Implications for future practice

Given the limited available evidence in relation to experiences and effective techniques for clinical trials management, this study provides valuable information to help to inform future research design and conduct. Although the findings are derived from a single study, the size and composition of the trial team from which feedback was collected has ensured a diverse range of feedback in relation to trial management techniques, of which many comments would apply more broadly across other trial types and settings. The adoption of key principles as outlined in earlier research [2, 4, 7] and their further development in the context of OCTET led to the overall success of the trial.

The inclusion of two trial managers ensured consistent communication throughout the trial and, therefore, it is suggested that including multiple managers or coordinators should be considered particularly when designing future complex or multi-centre trials. When compiling a study management team an important characteristic, which should not be underestimated or overlooked, is that of staff personability as this can be critical in promoting an effective and efficient study-wide team. Employment regulations prevent staff selection solely on the basis of high-quality interpersonal skill and so interpersonal skills training opportunities should be offered by institutions and considered as a part of wider professional development.

Research design should ensure that project burdens are appropriately balanced against the existing responsibilities of any involved parties (e.g. NHS or third party sites) as this will ensure an efficient trial which can be easily executed and monitored. Identifying expectations of all involved parties at the earliest possibility is imperative to ensure a cohesive team and so links between involved parties are established early during study set-up, and built upon further as the trial progresses.

Procedures and resources (e.g. procedures, database) should be compiled from the outset, through collaboration with those who will be working with or to these documents to ensure that any documentation is intuitive to those delivering the research. Procedures should also consider and promote both patient and researcher safety, particularly when face-to-face visits are to be completed. Focussing on the safety of both groups promotes a caring ethos which can subsequently help to facilitate positivity and this in turn impacts favourably on both the execution and monitoring of the study.

## Conclusions

This study makes a valuable contribution to the limited available evidence drawing on real-life experiences of executing a research trial. By utilising qualitative methods to elicit feedback from individuals involved in a multi-centre clinical trial this has captured diverse opinion on clinical trials management acceptability. Communication, positivity and clear processes and resources are crucial for both executing and monitoring a trial successfully and so trial managers should consider developing and including clear processes, resources and positive, open communication pathways when setting up a study. Further insight of the experiences of individuals involved in research studies, and the continued sharing of effective techniques, will help to further evolve efficient trial management in the future.

## Additional files

Additional file 1: Learning from OCTET Questionnaire. (DOCX 14 kb)

Additional file 2: COREQ Checklist of items for reporting of qualitative research. (PDF 491 kb)

## Abbreviations

cCBT: Computerised cognitive behavioural therapy
DMEC: Data Monitoring and Ethics Committee
DMEC/TSC: DMEC or TSC member
NHS: National Health Service
NIHR: National Institute for Health Research
OCTET: Obsessive Compulsive Treatment Efficacy Trial
PWP: Psychological wellbeing practitioner
R: Researcher questionnaire
RCT: Randomised controlled trial
RFG: Researcher focus group
SL: Site lead
TSC: Trial Steering Committee
UK: United Kingdom
YBOCS: Yale Brown Obsessive Compulsive Scale
