# Supplementary material for: Learning from OCTET – exploring the acceptability of clinical trials management methods
Source: Trials. 2018 Jul 13;19:378. doi: 10.1186/s13063-018-2765-6 (PMC6045834; doi:10.1186/s13063-018-2765-6)
Supplement: Supplementary file 1 — Learning from OCTET Questionnaire. (DOCX 14 kb) [file 13063_2018_2765_MOESM1_ESM.docx]

**Learning from your experiences**

We would like to gather some feedback about various aspects of the trial including: The management of the trial, the trial procedures, trial training, your involvement and experience, what could be done better.

If you would like to provide any feedback in relation to the below questions this would be very much appreciated. We encourage you to be as honest as possible – we want your comments both good and bad. We will then collate the comments from all trial team members and will look to provide a report or paper on the learning from OCTET in due course.

**What has gone well so far?**

**What hasn’t gone so well?**

**What would have helped to make your involvement in the trial more positive?**

**Could any aspect of the trial have been organised or completed more efficiently?**

**Were your training needs met? Would you have preferred that the training be conducted in a different way?** *– Please consider any training event(s) you attended in relation to OCTET.*

**Was there anything that we provided that you did not expect?**

**How does your experience compare to other trials you have worked on/other working environments?**
